# Automated quantification of epicardial adipose tissue in cardiac magnetic resonance imaging

**DOI:** 10.1186/1532-429X-16-S1-P363

**Published:** 2014-01-16

**Authors:** María Luaces, Alexandra Cristobal-Huerta, Juan A Hernandez-Tamames

**Affiliations:** 1 Cardiology, Hospital Clínico San Carlos, Madrid, Madrid, Spain; 2 Electronic, Universidad Rey Juan Carlos, Mostoles, Madrid, Spain

## Background

The prevalence in obesity has been doubled in the last 20 years. Obesity exponentially increases the relative risk of cardiovascular disease and shortens life span. Epicardial adipose tissue (EAT) has emerged as an independent predictor of high cardiometabolic risk. Cardiac magnetic resonance could potentially be a robust tool for absolute fat quantification. Quantification of EAT has usually been performed by manual segmentation. However, manual segmentation is observer biased and time consuming. The aim of this work is to develop an automated method for quantification of EAT taking into account "a priori" anatomical information.

## Methods

Ten morbidly obese patients, without preexisting heart conditions, (8 women, 2 men, mean IMC: 46.38 ± 5.41 Kg/m2) were included in the study. Cardiac-MR was performed with a Signa 1.5T GE MR Scanner. Scout images from the vertical and horizontal long axis and a stack of 2D short axis cine SSFP were acquired. Only five central images were chosen to be quantified to guarantee the signal homogeneity across subjects and avoiding artifacts in extreme slices. Endocardial and epicardial boundaries for both ventricles, as well as the EAT, were manually delineated by an expert cardiologist, and used as "gold standard" to appraise the quality of the proposed automated quantification. The automated method consists of the delimitation of the region of interest (ROI), heart and pericardial fat, from the image through the subtraction of the cine images followed by a Laws filter to detect the EAT and the endocardial boundary. To extract and quantify the cardiac muscle, histogram thresholding was applied to the ROI. In the case of the EAT, it was differentiated from the paracardial fat by means of a curvature analysis. K-cosine algorithm was used for this purpose. Since heart size varies subject by subject, the ratio between the EAT volume and the myocardium was proposed as a new normalized measure for study group. Dice similarity coefficient, showing the overlapping between segmentations, and a t-test were used as metrics to compare manual and automated segmentations.

## Results

The mean Dice Similarity Coefficient for the segmentation of the muscle and EAT was 85.12 ± 3.02% and 61.16 ± 13.97%, respectively (> 70 is considered acceptable). For the ten patients, the ratio obtained was 0.39 ± 0.20% and 0.52 ± 0.26%, for the manual and automated quantification, respectively. The paired t-test provides no significant difference between the different methods for the ratio (p = 0.2, uncorrected). In terms of time consuming, the manual method takes 13 minutes versus 2.5 minutes for the automatic.

## Conclusions

This study presents an automated epicardial adipose tissue tool for MR. Preliminary results shed no significant differences between methods with a remarkable time saving when the automatic method is used. Further work would include: improvement of the quantification accuracy and to develop a friendly graphical user interface for cardiologists.

## Funding

Fund for Health Research. Carlos III Health Institute. Ministry of Economy and Competitiveness of Spain. (PI09/0871 and PI09/2428).

**Table 1:** The table shows the mean values for the Dice similarity coefficient and the ratio obtained by the manual and automatic segmentation.

	Manual segmentation	Automatic segmentation	P-value
Dice Coefficient	85.12 ± 3.02%	61.16 ± 13.97%	

Ratio	0.39 ± 0.20	0.52 ± 0.26	0.2

**Figure 1:**
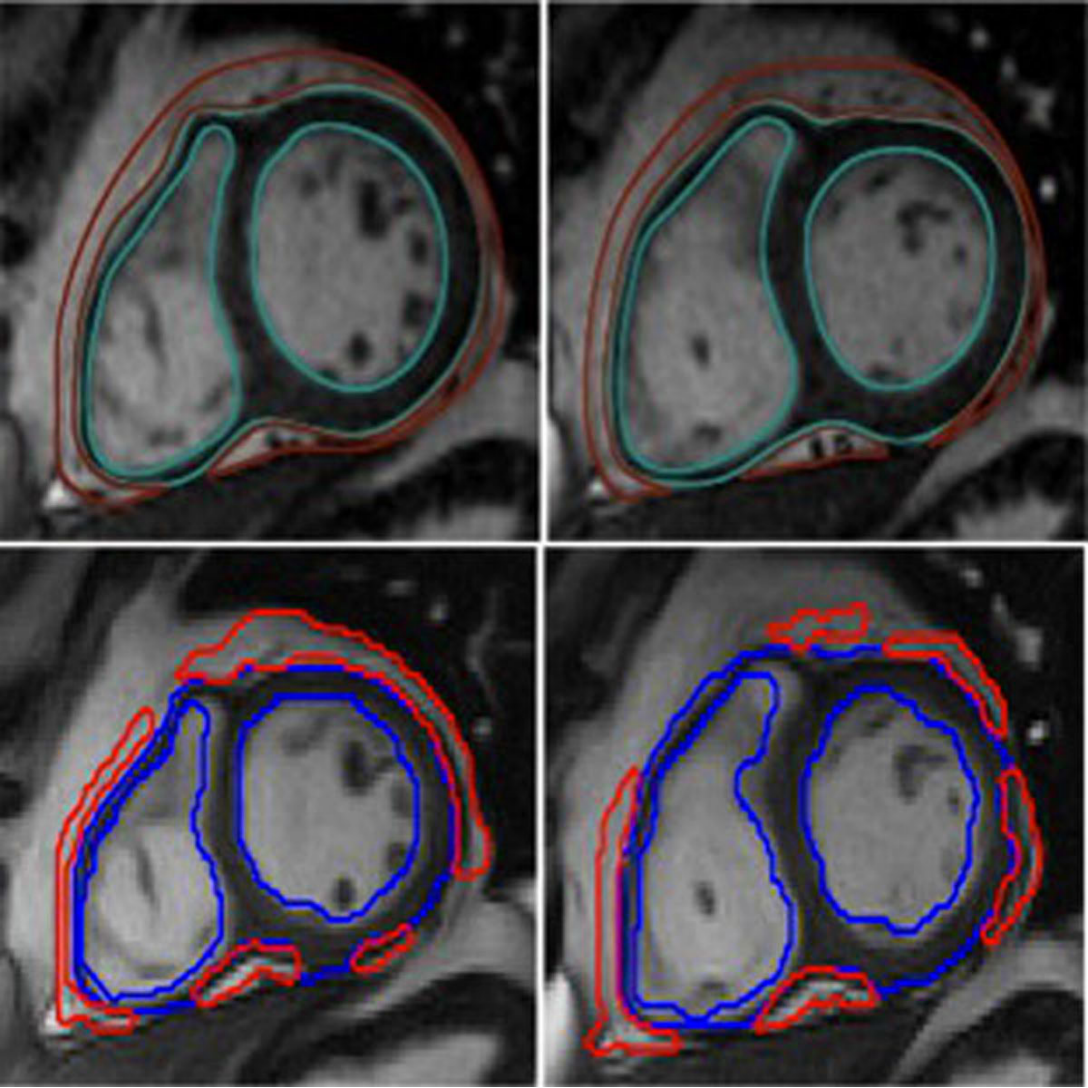
**The figure shows two images of the manual segmentation (above) and the automated segmentation (below) from one subject**. Green/blue contours, correspond to the cardiac muscle and the red ones to the epicardial fat.

